# Selective protected state preparation of coupled dissipative quantum emitters

**DOI:** 10.1038/srep16231

**Published:** 2015-11-09

**Authors:** D. Plankensteiner, L. Ostermann, H. Ritsch, C. Genes

**Affiliations:** 1 Institut für Theoretische Physik, Universität Innsbruck, Technikerstrasse 21a, A-6020 Innsbruck, Austria

## Abstract

Inherent binary or collective interactions in ensembles of quantum emitters induce a spread in the energy and lifetime of their eigenstates. While this typically causes fast decay and dephasing, in many cases certain special entangled collective states with minimal decay can be found, which possess ideal properties for spectroscopy, precision measurements or information storage. We show that for a specific choice of laser frequency, power and geometry or a suitable configuration of control fields one can efficiently prepare these states. We demonstrate this by studying preparation schemes for strongly subradiant entangled states of a chain of dipole-dipole coupled emitters. The prepared state fidelity and its entanglement depth is further improved via spatial excitation phase engineering or tailored magnetic fields.

Ensembles of effective two-level quantum emitters consisting of single atoms, ions, or defects in solids are employed ubiquitously in quantum optics and quantum information^1^. They are the basis for precision spectroscopy or atomic clock setups, as well as for experiments testing fundamental concepts of quantum physics or implementations of the strong coupling cavity QED (quantum electrodynamics) regime^2^,^3^. In the absence of direct particle-particle interactions, larger ensembles allow for faster, more precise measurements^4^ via a scaling of the effective single photon to matter coupling strength *g* by a factor 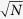 (with system size *N*) and a reduction of the quantum projection noise (by 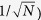
5,6.

For any precise measurement one has to externally prepare, control and measure the particle dynamics. Hence, the emitters are almost unavoidably coupled to their environment. A suitable theoretical framework to model such experiments is open system dynamics with a coupling to a fluctuating thermal bath. At optical frequencies this can often be approximated by the zero effective temperature electromagnetic vacuum field^7^,^8^. Still, extra perturbations by a thermal environment and background gas collisions cannot be avoided.

In a laboratory experiment the particles need to be confined in a finite spatial volume that can be addressed by laser beams. Thus, increasing particle numbers will lead to higher densities, where direct particle-particle interactions as well as environmentally induced collective decoherence can no longer be neglected. For optical transition frequencies a critical density is conventionally assumed at the point where the average particle separation is of the order of an optical wavelength^9^. Above this limit vacuum fluctuations tend to become uncorrelated and decay becomes independent. However, recent calculations have shown that collective states can exhibit superradiance and subradiance even at much larger distances^10^ as long as the bandwidth of the emission is small enough.

In many typical configurations and in optical lattices in particular, the particle-particle interaction is dominated by binary dipole-dipole couplings, with its real part inducing energy shifts and its imaginary part being responsible for collective decay^11^,^12^. Generally, this interaction is associated with dephasing and decay. However, recently it has been found that under special conditions also the opposite can be the case and these interactions can lead to a synchronization^13^ or even a blockade of the decay^14^.

Often times it is assumed that while such states exist, they cannot be prepared by lasers as they are strongly decoupled from the radiation fields. However, it was recently proposed that individual instead of overall addressing of the atoms can push the many particle system to evolve towards subspaces protected from decay or dephasing^15^. When applied to Ramsey spectroscopy such states have been shown to exhibit frequency sensitivities superior even to those obtained from non-interacting ensembles^16^. However, apart from special cases with an optimal lattice size and excitation angle, it is not so obvious how to implement such precise a control.

In this work we highlight the surprising fact that interaction induced level shifts can be used to aid in preparing such states. In many cases the magnitude of the shifts a state experiences and its lifetime are tightly connected allowing one to identify and address interesting states via energy resolution. As a generic ensemble we particularize to a 1D regular chain of quantum emitters coupled by dipole-dipole interactions with a tunable magnitude (by varying the interparticle separation). Collective coupling to the vacuum leads to the occurrence of subradiant as well as superradiant excitonic states^10^. In particular, the subradiant states should prove extremely useful for quantum information as well as metrology applications as they exhibit robust, multipartite quantum correlations. As mentioned above, the atoms’ interactions provide a first handle for target state selection as they lead to energy resolved collective states. Furthermore, using a narrow bandwidth laser excitation matched to the target states both in energy and symmetry allows for a selective population transfer from the ground state via an effective Rabi *π*-pulse.

In many cases, however, the required phase structure of the target state is not compatible with the excitation laser phase so that only a very weak coupling can be achieved. On the other hand, increasing the laser power reduces spectral selectivity by an unwanted addressing of off-resonant but strongly coupled states. Hence, to address a larger range of states of practical interest, we also propose and analytically study new methods of phase imprinting via a weak spatial magnetic field gradient. The small relative phase shifts increase the effective coupling to groups of emitters via a nonuniform phase distribution. With this method any state may acquire a finite laser coupling to the ground state via the magnetically induced level shifts resulting in an efficient population transfer with a minimal compromise on lifetime.

The considered setup is a chain (see Fig. 1a) of *N* identical two-level systems (TLS) with levels 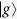 and 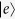 separated by a frequency of 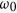 (transition wavelength *λ*_0_) in a geometry defined by the position vectors 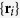 for 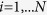. For each *i*, operations on the corresponding two-dimensional Hilbert space are written in terms of the Pauli matrices 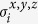 and raising/lowering operators 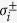 connected via 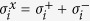, 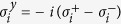 and 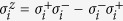. The complete Hamiltonian describing the coherent dynamics is


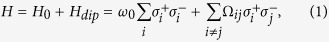


where *H*_*0*_ is the free Hamiltonian and has degenerate energy levels (degeneracy 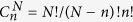 for level *n*) ranging from 0 for the ground state to 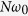 for the highest excited state. The second term *H*_*dip*_ describes interactions between pairs of TLS which can be induced either by an engineered bath (such as a common, fast evolving optical cavity field) or by the inherent electromagnetic vacuum. We denote the couplings between emitters *i* and *j* by 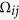 and particularize to the case of a free-space one dimensional equidistant chain of TLS with small interparticle distances *a* such that 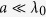 (as depicted in Fig. 1a).

For the sake of simplicity, we use dipole moments perpendicular to the chain for all numerical computations. To a good approximation, in the limit of 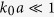, the nearest-neighbor (NN) assumption can be used (such that 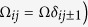 and exact solutions in the single-excitation manifold can be found^17^. Within this subspace and approximation, the Hamiltonian assumes the form of a tridiagonal symmetric Toeplitz matrix with 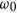 on the diagonal and Ω above and below the diagonal. The solutions are readily available^18^ with eigenvalues 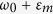 for an index *m* running from 1 to *N*, where 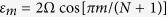 are the dipole-induced energy shifts. The corresponding eigenstates of the Hamiltonian are then


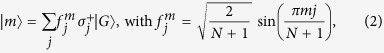


where we used 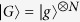.

Spontaneous decay via a coupling to the free radiation modes in the evolution of the system can be included in a generalized Lindblad form^8^,


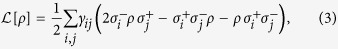


where the *γ*_*ij*_ denote collective damping rates arising from the coupling to a common radiation field. These rates also strongly depend on the atomic distance *a* with two prominent limiting cases of 
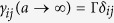
 (independent emitters limit) and 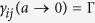 (the Dicke limit^19^). In general, one can perform a transformation of the Liouvillian into a new basis by diagonalizing the *γ*_*ij*_ matrix. This procedure leads to a decomposition into *N* independent decay channels with both superradiant (>Γ) and subradiant (robust) decay rates (<Γ)^16^. Note, however, that the states corresponding to these channels generally do not coincide with energy eigenstates of the Hamiltonian, so that we cannot reduce the system dynamics to simple rate equations.

## Results

### Selective state preparation

#### Tailored coherent excitation

As mentioned above, our dipole coupled systems possess states with a large range of radiative lifetimes and energy shifts. Depending on the desired application particular states can be highly preferable over others. In a first straightforward approach we now illustrate that in principle it is possible to access a desired collective state simply by a selective coherent driving with a properly chosen amplitude and phase for each TLS. This is described by the Hamiltonian


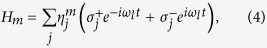


with a suitably chosen set of 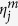. For a targeted eigenstate in the single-excitation manifold, some analytical insight on how to choose these amplitudes can be gathered from the state’s symmetry. For energy eigenstates this can be found quite reliably within the NN approximation^20^. In an equidistant finite chain our calculation suggests the following choice of driving fields at laser frequency 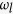,


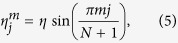


chosen to fit the symmetry of a target state 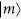.

**Figure 1:**
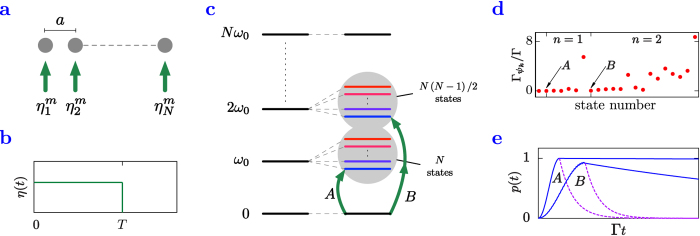
Selective state preparation procedure. (**a**) A chain of 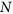 closely spaced quantum emitters (separation 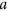 with 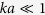, 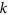 being the laser wave number) are individually driven with a set of pumps 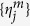. (**b**) The lasers are turned on for a time 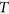, optimized such that an effective 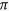-pulse into the desired subradiant target state is achieved. (**c**) Level structure for the 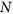 systems where the 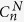-fold degeneracy of a given 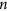-excitation manifold is lifted by the dipole-dipole interactions. The target states are then reached by energy resolution (adjusting the laser frequency) and symmetry (choosing the proper 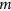). (**d**) Scaling of the decay rates of energetically ordered collective states starting from the ground state (state index 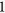) up to the single- and double-excitation manifolds for 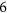 particles at a distance of *a* = 0.02*λ*_0_. The arrows identify the decay rates for the lowest energy states in the single (A) and double (B) excitation manifolds. (**e**) Numerical results of the time evolution of the target state population for *N* = 6 and *a* = 0.02 *λ*_0_ during and after the excitation pulse. Near unity population is achieved for both example states A (where we used *η* = 0.53 Γ) and B (for *η* = 2.44 Γ) followed by a subradiant evolution after the pulse time 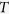 shown in contrast to the independent decay with a rate 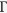 (dashed line).

The selectivity of the excitation process can be further improved by an *energetically resolved excitation* of a given state 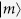 by a proper choice of the laser frequency 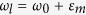 and its bandwidth. This is possible due to the interaction induced level splitting from 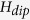 (as depicted in Fig. 1c). Indeed, in perturbation theory and in a frame rotating at 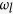 the evolution of the system starting from the ground state up to a normalization factor leads to


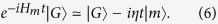


The success of the corresponding process is illustrated in the sequence of plots in Fig. 1, where the *m* *=* *N* state with *n* *=* 1 is considered (target state A) and accessed via the combination 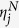 of pumps lasting for a duration *T*.

Numerical simulations were performed on a six-atom chain with driving strength 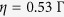 at an interatomic separation of 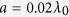. The time for which the pumps are switched on is 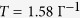 which is considerably shorter than the time scale governed by the decay rate of 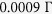 of the target state. The resulting dynamic is an effective *π*-pulse (efficiency of 99.94%) flipping the population into the state 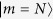 followed by an extremely slow decay, indicating the robustness of the target state (as seen in curve A of Fig. 1e).

It is, of course, desirable to target higher excitation manifolds as well. In the absence of analytical expressions or good approximations for the target states, we employ phases that yield maximal asymmetry, i.e. 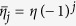 for any *j* *=* 1,...,*N*. Such a driving can be expected to address collective states, where the fields emitted by any two neighboring particles interfere destructively^14^ (similar to a previously investigated mechanism^15^). Numerical simulations show that the resulting collective states indeed exhibit the lowest energy shifts of the targeted manifold and can be expected to be long lived. The resonance condition for a specific state 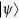 within the manifold *n* is 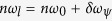, where 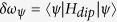. As an illustration, the curve B in Fig. 1e shows an almost perfect efficiency (98.36%) two-photon 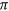-pulse allowing for a population transfer to the longest-lived collective state in the second excitation manifold of 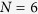 emitters separated by 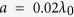. The chain was driven with a strength of 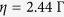 for a time 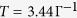, which again is significantly shorter than the natural time scale given by the target state decay rate of 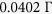.

Let us add a comment on the practical implementation of such an addressing. In typical current experimental configurations for clocks based on 1D magic wavelength lattices^21^,^22^ the atoms are very close and hardly allow for an individual direct particle addressing. One is largely limited by a quasi plane wave driving, which typically addresses all particles with equal intensity. If the pump light is applied perpendicularly to the trap, the evolution is governed by a symmetric Hamiltonian 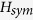, obtained from equation (4) with an equal pump amplitude 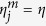 for any 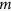 and 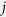. A laser excitation from the ground state into the state 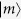 is connected to the coupling amplitude 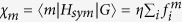, which yields


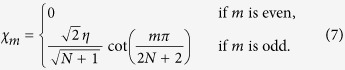


We will refer to states with even 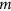 as *dark states* as they cannot be accessed by the laser excitation and call the remaining ones *bright states*^14^. In the limit of large atom numbers 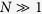, it is of interest to investigate the two cases, where 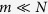 and 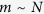, for states at the top/bottom of the manifold. In the first case, the function for the driving yields 
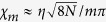
, whereas in the other case we have 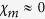.

Note, that sometimes geometry can change this behavior. For a 1D string of equidistant emitters illumination at a chosen angle of incidence and polarization leads to a designable phase gradient of the excitation amplitudes. The situation becomes even more complex for a 3D cubic lattice, where the phases also differ in the different lattice planes. As a lucky coincidence, a perpendicular plane illumination at the clock frequency in a magic lattice for Strontium (Sr) targets an almost dark state. This leads to subradiance and in principle allows for a spectral resolution better than the natural linewidth^23^. In not so favorable cases one could also think of a specific lattice design to facilitate a tailored dark state excitation.

#### Radiative properties

In order to be useful resources for quantum information applications, target states should exhibit *robustness* with respect to the environmental decoherence. To identify states of minimum decay rate, we scan through the eigenstates 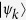 of the Hamiltonian 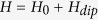 (for 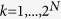) and compute their decay rates 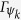 (see section Methods below). We find that generally, for a given manifold, the energetic ranking of the states closely indicates their robustness to decay (as illustrated by the color-coding in Fig. 1c) ranging from blue for subradiant states to red for superradiant states. This is due to the fact that both radiation and energetic shifts are strongly dependent on the symmetry of the states. In Fig. 1d, for 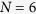, we plot the decay rates of the collective states in the first (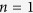) and second (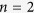) excitation manifold arranged as a function of their increasing energy corresponding to the level structure of Fig. 1c. Superradiant states are found at the upper sides of the manifolds while the ideal robust states lie at the bottom. In Fig. 1d, the arrows indicate the optimal decay rates in the single- (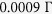) and double-excitation manifolds (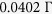) corresponding to target states A and B whose population evolution is depicted in Fig. 1e.

Within the single-excitation manifold, an analytical expression for the decay rate of a state 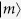 can be found as 
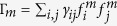
. For small distances the state 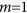 (upper state) is superradiant, whereas states at the bottom of the manifold 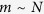 exhibit subradiant properties. In the Dicke limit where 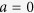 we have 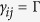 for any 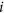 and 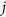, and we can compute 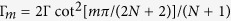 for 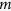 odd and 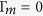 for 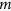 even. Note, that in this particular limit, these are the same conditions as for the darkness and brightness of a state. For large numbers of emitters, we recover the expected superradiant scaling with 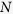 for the state with 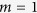, i.e. 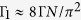. On the other hand, large 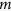 yield a decay rate of 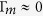 (perfect subradiance) in the same limit.

There are two important conclusions from these results: i) since in the considered limit the decay rate of the superradiant state 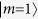 scales with 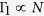, whereas its driving is 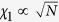, driving this state becomes more difficult with increasing atom number due to the reduced time-scale and ii) if the number of atoms is not too large, 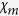 will remain finite, while 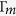 already indicates vast subradiance due to its scaling-down with 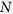. Hence, there are robust states that remain bright, i.e. they can be driven directly even though the driving is not matched to their symmetry.

### Accessing dark states via magnetic field gradients

**Figure 2:**
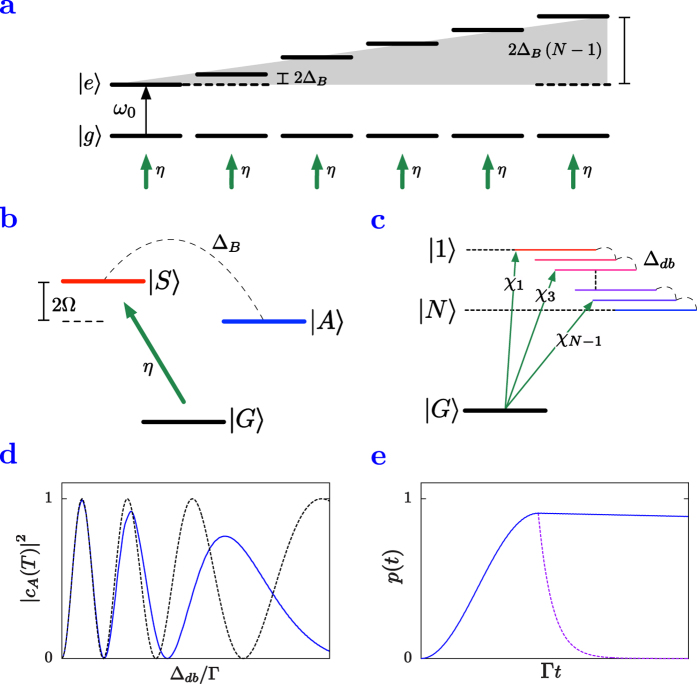
Coupling to dark states via a magnetic field gradient. (**a**) Linearly increasing level shifts along the chain occuring in the presence of the magnetic field gradient. (**b**) Illustration of the level structure and indirect dark state access for two coupled emitters. While symmetry selects the state 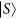, off-resonant addressing combined with bright-dark state coupling of strength 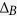 allows for a near-unity population transfer into the state 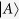. (**c**) Dynamics in the single-excitation manifold of 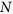 coupled emitters where symmetric driving reaches the bright states with amplitudes 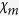 while the magnetic field couples neighboring dark and bright states. (**d**) Plot of the asymmetric state population for the two-atom case as a function of the increasing magnetic field (solid line) compared to the steady-state approximation (dashed line) at numerically optimized time *T* = 16.19 Γ^−1^, with parameters *η* = Γ and *a* = 0.05 *λ*_0_. (**e**) For a chain of *N* = 4 emitters, a 91%-efficient 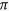-pulse to the most robust state can be achieved as demonstrated in the population evolution plot. The separation is chosen to be *a* = 0.025*λ*_0_, while *η* = 40 Γ and numerical optimization is employed to find Δ_*B*_ = 0.98 Γ.

The direct symmetric driving with 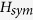 allows access to bright states only. Given that nearby dark states can conceivably be more robust, we now employ a progressive level shifting mechanism that allows for a coupling between bright and dark states. This is achieved by subjecting the ensemble to a magnetic field with a positive spatial gradient along the chain’s direction. The increasing energy shift of the upper atomic levels (as depicted in Fig. 2a) plays a role similar to the individual phase imprinting mechanism described previously. For each particle the shift of the excited level induces a time-dependent phase proportional to the value of the magnetic field at its position. We demonstrate the mechanism for a particular two-atom example, where indirect near unity access to the dark subradiant asymmetric collective state is proven and extend it to the single-excitation manifold of 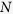 atoms.

#### Two-atom case

The eigenstates of the Hamiltonian 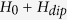 are 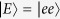, 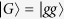 and in the single-excitation subspace 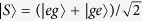 and 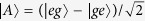. The symmetric state 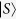 is superradiant (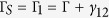) and bright, directly accessible via symmetric driving with strength 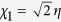. The asymmetric state 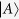, on the other hand, is subradiant (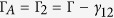) and dark. Indirect access can be achieved by shifting the second atom’s excited state by 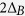 (see schematics in Fig. 2b), where 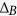 is tunable and quantifies the per-emitter shift for a given magnetic field amplitude. We first analyze the dynamics in the absence of decay by solving the time-dependent Schrödinger equation governed by the Hamiltonian 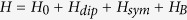, where 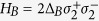. We reduce the dynamics to three states, and assume a quasi-resonant Raman-like scheme where the population of  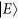 is at all times negligible. An effective two-level system arises (between the ground state and the asymmetric state; see section Methods below) and the resonance condition can be identified as


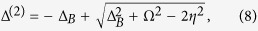


with an effective Rabi frequency of


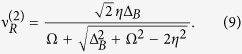


To fulfill 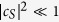, we need to restrict the driving to a parameter regime where 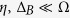. A scan over the magnetic field is performed and the exact numerical results for the asymmetric state population are plotted in Fig. 2d against the adiabatic solution showing near unity population transfer for an optimized 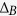. Further restrictions are imposed when decay is considered. These stem from the fact that the coherent process described by 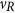 should be faster than the incoherent one characterized by 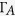. For close particles, the ability to tune the distance ensures that the scaling down of 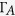 is very fast and the above conditions are readily fulfilled. For the particular example illustrated in Fig. 2d we chose 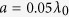, resulting in 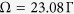, 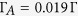. The 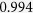 population is reached at 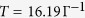, which is very close to the theoretical estimate of 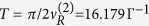 obtained from the adiabatic solution under the assumption of a 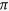-pulse transferring the population to the target state.

#### Many-atom case

For a chain of 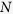 atoms, we consider the progressive shifting of excited levels along the chain depicted in Fig. 2a. This is realized by the application of a magnetic field with a constant gradient and is described by the Hamiltonian 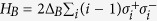. Let us consider a dark state 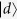 (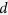 even) and the bright state 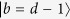 immediately above. Their coupling via 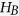 is quantified by 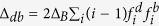, as shown in Fig. 2c.

We develop a protocol where direct off-resonant driving into the bright state (amplitude 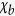) combined with a coupling between the bright and dark states via the magnetic field leads to an almost unity population transfer into the dark state. Given a sufficient energy separation, the problem can be reduced to solving the time-dependent Schrödinger equation for the three coupled state amplitudes 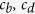 and 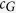. Following the same adiabatic approximation as in the two-atom case we reduce the general dynamics to an effective two-level system between the states meant to be connected by an effective 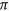-pulse, i.e. 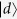 and 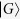. The generalized resonance condition (with 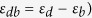 reads


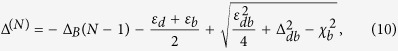


and was obtained in the limit where the coupling of the dark state to the other adjacent bright state 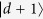 was neglected owing to the relation 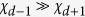. The effective transition rate between the ground state and the state 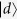 is


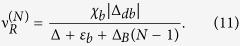


The addition of decay imposes a new constraint on the timescale of the process, i.e. 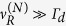, required to ensure near unity population in the dark state. The fulfillment of this condition depends on the individual system under consideration. As an illustration of the procedure, Fig. 2e presents the targeting of a robust dark state in the single excitation manifold of four particles. Note, that the numerical results are performed in an exact regime beyond the NN approximation and are in excellent agreement with our conclusions obtained from the NN treatment.

## Discussions

### Entanglement properties

**Figure 3:**
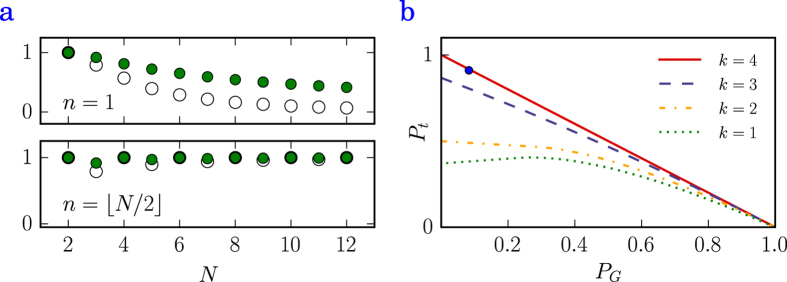
Entanglement properties. (**a**) Comparison of the numerically computed von Neumann entropy (empty circles) of the reduced density matrix of the chain minimized over the atom index and the analytical expression for the entropy of the Dicke state (green circles), both for excitations *n* = 1 and 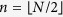 as a function of the atom number 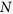 at distance *a* = 0.1 *λ*_0_. (**b**) Depth of entanglement of the subradiant four-atom state (blue dot) prepared by the magnetic field gradient scheme (see Fig. 2e). It clearly lies above the *k* = 3 boundary indicating four-atom entanglement. The 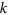-atom entanglement boundaries of the target state population 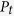 as a function of the ground state population 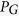 have been computed for the corresponding target state of a four-atom chain at distance *a* = 0.025 *λ*_0_.

To justify the usefulness of collective states for quantum information purposes, we employ the von Neumann entropy to analyze their entanglement properties. More specifically, we compute the von Neumann entropy of the reduced density matrix 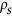 of a single two-level emitter (showing the degree of its bipartite entanglement with the rest of the system) defined by 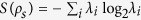, where 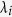 is the 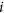-th eigenvalue of 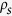 and 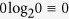. We furthermore minimize the set of values for all atoms to obtain a lower bound on the entanglement contained in the system. We compare the numerical results to the single-atom entropy of the symmetric Dicke state 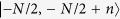
19. For these particular states the entropy is maximized if the number of excitations in the state is 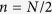. It follows that it is highly desirable to drive the system into robust states as close as possible to 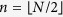 excitations (where 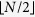 is the largest integer smaller or equal to 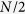), since this manifold contains the most entangled state. A comparison of the exact numerical data and the analytical expression for the entropy is shown in Fig. 3a.

Another way to characterize the entanglement of the prepared state is to investigate their *depth of entanglement*^24^,^25^, which does not quantify the entanglement itself but rather shows how many atoms of an ensemble are involved in the present entanglement. This measure has been used in recent experiments^25^,^26^ since it is a readily measurable quantity. The depth of entanglement is computed as follows: given an 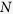-atom target state in which an arbitrary number of said 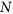 atoms is entangled, we compute the limit of how much population one can drive into this state such that the resulting density matrix 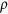 remains separable into a subset of density matrices that exhibit no more than 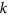-atom entanglement (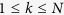). This may be done by numerically maximizing the target state population 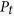 as a function of the ground state population 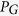 for different 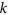. The boundaries themselves indicate how many atoms need to be entangled in order to prepare the pure target state, i.e. the boundary where the target state population is maximized to 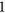 corresponds to the number of atoms entangled in the (pure) target state. If a general prepared state has a target and ground state population such that the corresponding data point lies on or above the 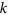-atom boundary, more than 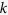 atoms are entangled.

Obviously, for the pure target states considered in the above computation all atoms contribute to the entanglement, since otherwise the minimal von Neumann entropy as shown in Fig. 3a would be zero. For a more interesting result, we can compute the depth of entanglement in order to demonstrate the efficiency of the driving procedure using a magnetic field gradient as in Fig. 2e. From Fig. 3b, where all boundaries have been plotted for the considered subradiant four-atom state, it is clear that the prepared state shows all-atom entanglement as the corresponding data point lies far above the boundary for three-atom entanglement.

### Implementation considerations

The proof-of-principle technique presented above has been particularized on a specific generic system of emitters in an equidistant chain. The choice is natural since the electromagnetic vacuum provides a simple example for both collective dispersive and dissipative dynamics. To exemplify a possible realization we consider a particular system^27^ where bosonic Sr atoms are trapped in a magic wavelength optical lattice at separations of *a* *=* 206.4 nm. The working transition is at 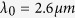, between the 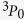 and 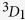 electronic states. This amounts to a ratio of 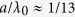 which allows for an operation in the regime targeted by our scheme. The corresponding single atom decay rate is at the order of 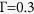 MHz and circularly polarized light can allow for transitions between states with a difference of 1 in magnetic quantum number. We have numerically investigated a system of 4 atoms in such a configuration and found a sizeable 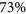 target state population for 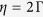 and 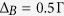, under the conditions of a relatively small level shift between the dark and bright state around 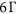 which does not allow for large driving powers. For further optimization of the efficiency of the target state preparation one could envision a modified setup where a trapping transition of smaller wavelength can be chosen that would most importantly allow for better state separation (owing to larger dipole shifts). The corresponding magnetic field gradient required to produce the considerable 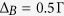 shift on a distance of 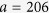 nm is around 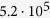 G/m, not far from state-of-the-art values achievable in high magnetic field gradient magneto-optical trap experiments^28^,^29^. Of course, there are many detrimental practical effects that can seriously limit the above technique such as light-assisted collision loss. We envision the extension of the described technique to systems where both the coherent and dissipative particle-particle interactions can be suitably tailored. For example, the same kind of dipole-dipole Hamiltonians can occur in 3D lattices of polar molecules^30^ or between two different color NV centers in diamonds^31^.

### Conclusions

Direct particle interactions are typically detrimental and limiting in precision measurement applications. Here, we have presented some specific opposite examples, where the *collective* nature of the decoherence combined with the coherent binary dipole-dipole interactions is used as a new resource for the controlled and efficient preparation of specially selected states. The excitation scheme can be tailored to address target states exhibiting both entanglement as well as robustness against decay. As a generic example we studied the case of a one-dimensional system of tightly spaced equidistant quantum emitters. Already the inherent dipole-dipole coupling allows for a targeted state preparation technique via energy selection. The performance of the excitation can be enhanced additionally via the *continuous* application of a spatially increasing magnetic field. The general principle of such a phase imprinting technique is potentially applicable in many specific environments such as optical lattices or atoms and ions localized within one or more common optical cavity modes^32^,^33^, NV-centers or superconducting qubits coupled to CPW transmission lines or resonators^34^,^35^.

## Methods

### Decay rate of the states

In order to arrive at an analytical expression for the decay rate of an eigenstate 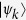 of the Hamiltonian in equation (1), we consider the homogeneous part of the differential equation of the corresponding density matrix element that arises from the master equation. The solution of this differential equation yields an exponential decay. The rate at which the state population decays may be written as


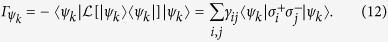


Note, that this is true only for states that contain one specific number of excitations, i.e. they are eigenstates of the operator 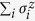. Obviously, this is fulfilled for eigenstates of the considered Hamiltonian. Equation (12) was used in order to compute the rates depicted in Fig. 1d and throughout the manuscript. For example, we used it in order to compute the decay rate of the eigenstates in the NN approximation 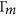.

### Subradiance and disorder

**Figure 4:**
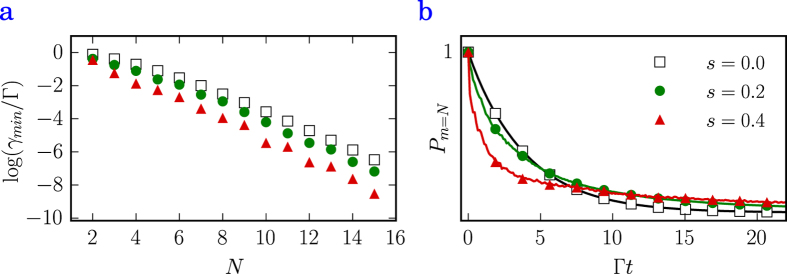
Subradiance and disorder. (**a**) Plot of the logarithm of the minimal eigenvalue of the decay rate matrix (matrix with entries 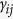) as a function of *N* at *a* = 0.4 *λ*_0_. for increasing levels of disorder (*s* = 0, 0.2, 0.4). (**b**) Decay of the 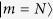 state as a function of time. In the presence of disorder (*s* = 0.2, 0.4) the short time and long time behaviors are fundamentally different. At short times, disorder can push the state towards faster decaying channels while decay inhibition due to disorder occurs at larger times.

Let us consider the influence of positioning disorder on subradiant properties of the target states. To mimic disorder we perturb an equidistant chain of 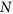 emitters (average separation 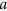) by introducing an uncertainty in each emitter position quantified by a defect parameter 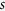 (normal distribution of variance 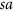). We then write the randomized matrix of decay rates and find the minimum decay channel without as well as in the presence of disorder of 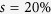 and 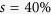. For the 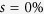 case, it has been shown^16^ that the minimum decay rate scales exponentially with 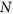 even for distances up to 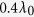, while the linear scaling with 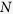 typical for superradiance is reached for 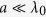 only. After averaging over 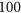 random configurations, we plot the logarithm of the minimal rates as a function of increasing 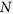 in Fig. 4a. As a somewhat surprising result, subradiance scales even better with 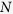 as the disorder increases. This might be understood as a destructive interference effect brought on by the cancelation of emitted photons stemming from the random positioning. As pointed out in previous investigations^16^, the states of low symmetry (as, for example, the 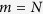 state) possess decay rates closest to the analytically derived minimal rate. We analyze the respective sensitivity of the state subradiance to disorder by initializing the system of 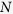 emitters in the 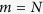 state and allow it to decay. The outcome is plotted in Fig. 4b and shows remarkable robustness of the disordered systems on a long time-scale. While on a short time-scale disorder pushes the considered state into faster decaying channels, the long time limit shows that the remaining population accumulates in the disorder-enhanced robust states.

For short time-scales, the state still decays slowly (subradiantly), however, the decay rate increases with growing disorder (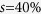). More remarkable, though, is the behavior the decaying states show for long time-scales, as the states subject to larger disorder become more robust than the unperturbed system. This is due to the fact that all population in the 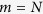 state that decays through more radiative channels have decayed at that point and only the most subradiant channel (minimal eigenvalue of the decay rate matrix) remains. As seen in Fig. 4a, this eigenvalue is even further reduced by disorder which explains the long time-scale behavior in Fig. 4b.

### Coherent dynamics with a magnetic field gradient

#### Two-atom case

To find the expressions in equation (8) and equation (9) we solve three coupled differential equations neglecting the population of the fully inverted state 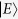 as far off-resonant for all times. In the collective basis, where any state may then be written as 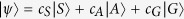, the equations are


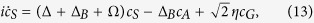



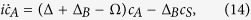



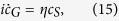


where 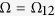 is the coherent interaction between the atoms and 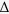 is the detuning between the atomic resonance frequency and the driving laser. For an efficient driving of 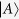 the population of the state 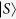 needs to be negligible which allows us to set a steady-state condition, namely 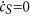 yielding the desired effective two-level system between 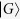 and 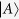.

#### Many-atom case

The same approach as in the two-atom case may be used to describe the dynamics in the single-excitation manifold for an arbitrary number of atoms in a chain. Given sufficient energy separation we may neglect all states but the ones we aim to address. We can indirectly address a dark state 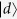 by driving the bright state 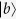 immediately above, which is coupled to the dark state by a magnetic field gradient. Neglecting all populations but 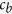, 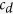, and 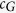 and their respective couplings via the magnetic field gradient, the investigation reduces to the equations


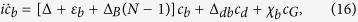



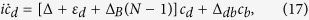



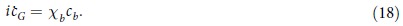


For an efficient driving of the dark state we may again invoke a steady-state condition on the bright state population 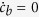. This, again, yields an effective two-level system between the ground and the dark state with resonance condition and Rabi frequency as displayed in equation (10) and equation (11), respectively.

### Von Neumann entropy

For a Dicke state an analytical expression for the von Neumann entropy of the reduced density matrix can be obtained. First, note that, since Dicke states are invariant under a permutation of the atoms, all reduced density matrices are identical. Hence, they all share the same von Neumann entropy for a given number of excitations 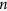. We may choose to reduce the full density operator 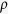 to the density matrix of the first atom in the ensemble, i.e. 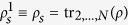 which yields a von Neumann entropy of


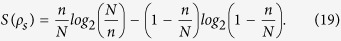


For the actual eigenstates of the Hamiltonian in Eq. (1) this computation needs to be done numerically. Furthermore, these states are not invariant under permutation of atoms and hence it is required to minimize the entropy with respect to the atomic chain index in order to find the lower bound.

### Depth of entanglement

The boundaries depicted in Fig. 3b were found by maximizing the target state population with the condition on the density matrix of the prepared state to contain no more than 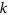-atom entanglement, i.e. 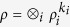 with 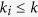 and at least one 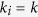. To compute the boundaries we generalized the algorithm that was previously used solely for the 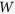-state^25^ to arbitrary states in the single-excitation manifold. For the computation of all boundaries we need to distinguish the two cases where 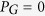 and 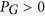. Considering a separable state (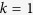), the boundary for 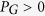 is found to be


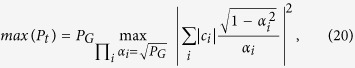


where 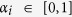 and 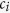 are the coefficients of the target state. For 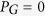 the maximization is much simpler, i.e. 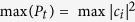, which is found by setting one 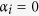 and the remaining coefficients 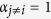. Note, that for both these and all following computations we neglect the symmetry of the state, i.e. the phases of the coefficients 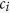 by using 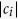. This is valid due to the invariance of entanglement under local unitary operations and necessary if we restrict the coefficients 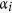 in the way we did.

For multiple-atom entanglement (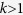) the matter of finding the corresponding boundary is no longer so simple. In order to find the maximum population, we assume maximally allowed entanglement in the prepared state. We split the prepared state into 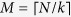 sets, where 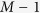 sets are 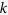-atom entangled and the remaining one is 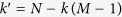-atom entangled. To find the maximum, one has to consider all possible positions of the 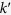-entangled state. If, for example, the 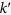-entangled state is at the last position, the population of the target state 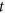 in the prepared state reads


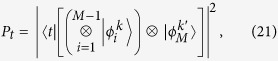


where


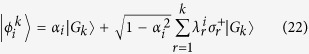


is a general non-separable state of 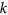 atoms in the single-excitation manifold. The state 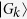 is the 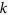-atom ground state and the coefficients 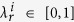 have to be normalized, i.e. 
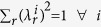
. One then has to maximize the target state population with respect to the coefficients 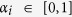 and 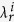 with the condition 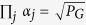. The number of these coefficients, however, grows vastly with the number of atoms, hence numerical computations are limited. For 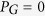 one can again choose one 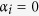 and all 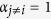.

Note, that all boundaries computed via this maximization only hold for pure states. In order to find the boundaries for mixed states we need to compute the convex hulls of the respective boundaries^25^. The 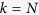 boundary is found when a perfect superposition between the ground and target state is reached.

In this work we considered the specific case of an exciton state of a four-atom chain. In that case, when investigating two-atom entanglement the permutation of the 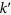-entangled state is rendered unnecessary since 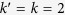. Unfortunately, this is no longer true for 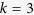, where we did have to account for all permutations.

## Additional Information

**How to cite this article**: Plankensteiner, D. *et al.* Selective protected state preparation of coupled dissipative quantum emitters. *Sci. Rep.* **5**, 16231; doi: 10.1038/srep16231 (2015).
